# Effectiveness of A Levonorgestrel-Releasing Intrauterine System Versus Hysteroscopic Treatment for Abnormal Uterine Bleeding in Women with Cesarean Scar Defects: A Systematic Review and Meta-Analysis

**DOI:** 10.3390/jpm15030117

**Published:** 2025-03-18

**Authors:** Athanasios Douligeris, Nikolaos Kathopoulis, Konstantinos Kypriotis, Dimitrios Zacharakis, Anastasia Prodromidou, Anastasia Mortaki, Ioannis Chatzipapas, Themos Grigoriadis, Athanasios Protopapas

**Affiliations:** 1 Minimally Invasive Gynecologic Surgery Unit, 1st Department of Obstetrics and Gynecology, National and Kapodistrian University of Athens, Alexandra General Hospital, 11528 Athens, Greece; nickatho@gmail.com (N.K.); kypriotisk@gmail.com (K.K.); ixatzipapas@yahoo.gr (I.C.); prototha@otenet.gr (A.P.); 2 Urogynaecology Unit, 1st Department of Obstetrics and Gynecology, National and Kapodistrian University of Athens, Alexandra General Hospital, 11528 Athens, Greece; dimzac@hotmail.com (D.Z.); aprodromidou@med.uoa.gr (A.P.); tgregos@yahoo.com (T.G.)

**Keywords:** cesarean scar defect, isthmocele, niche, levonorgestrel intrauterine device, abnormal uterine bleeding, postmenstrual spotting, hysteroscopic remodeling

## Abstract

**Background/Objectives**: To assess the effectiveness of the levonorgestrel-releasing intrauterine device (LNG-IUD) compared to hysteroscopic resection for managing women with symptomatic cesarean scar defects (CSDs). **Methods**: This systematic review and meta-analysis followed PRISMA guidelines. A comprehensive search of four electronic databases was conducted to identify studies comparing LNG-IUD with hysteroscopic management for symptomatic CSDs. Studies reporting outcomes of bleeding and spotting days and effectiveness rates were included. Quality assessment was performed using the ROBINS-I and RoB-2 tools. **Results**: Three studies involving 344 patients met the inclusion criteria. At 6 months, LNG-IUD use significantly reduced total bleeding days (MD −4.13; 95% CI: −5.17 to −3.09; *p* < 0.00001) and spotting days (MD 1.90; 95% CI: 0.43 to 3.37; *p* = 0.01) compared to hysteroscopic treatment. By 12 months, LNG-IUD demonstrated superior effectiveness (OR 3.46; 95% CI: 1.53 to 7.80; *p* = 0.003), with fewer total bleeding days (MD −5.69; 95% CI: −6.55 to −4.83; *p* < 0.00001) and spotting days (MD 3.09; 95% CI: 1.49 to 4.69; *p* = 0.0002). Approximately 50% of LNG-IUD users experienced amenorrhea within 1 year. **Conclusions**: LNG-IUD offers a minimally invasive and effective alternative to hysteroscopic resection for women with symptomatic CSD and no desire for future pregnancies. Its role should be considered in clinical practice, but further research is needed to validate these findings and define its long-term benefits and limitations.

## 1. Introduction

Cesarean scar defects (CSDs), or isthmoceles or niches, are focal myometrial defects occurring at the site of prior cesarean section incisions [1]. These defects, identified in approximately 56–78% of women with a history of cesarean delivery, are often associated with abnormal uterine bleeding, postmenstrual spotting, secondary infertility, and chronic pelvic pain [2,3]. Although the pathogenesis remains unclear, incomplete healing at the cesarean site, coupled with mechanical and inflammatory processes, is thought to contribute to the formation of these defects [4]. The rise in cesarean birth rates worldwide has made CSDs more relevant, underscoring the necessity for efficient management techniques [5].

The treatment of symptomatic isthmocele varies based on clinical presentation, reproductive desires, and residual myometrial thickness. Hysteroscopic resection is commonly used to excise fibrotic tissue and remodel the defect, offering effective symptom relief in suitable cases [6]. For women with thinner residual myometrium or higher reproductive risks, laparoscopic or vaginal CSD repair may be performed to restore uterine integrity. Although these surgical techniques are highly effective, they are inherently invasive, demand advanced surgical expertise, and carry a heightened risk of perioperative complications [7].

The levonorgestrel-releasing intrauterine device (LNG-IUD) is a minimally invasive option that has demonstrated potential in the treatment of symptomatic CSDs. By inhibiting endometrial development and decreasing vascularity through localized hormone release, the LNG-IUD lessens abnormal bleeding and spotting [8,9]. The evidence for its application is restricted to small-scale observational and comparative research, despite these suggested advantages [10]. Personalized medicine serves as a cornerstone of modern medical care, tailoring therapeutic choices to the specific needs of each patient. In the management of CSDs, factors such as reproductive desires, residual myometrial thickness (RMT), and accompanying symptoms play a pivotal role in guiding the selection of treatment. Both the LNG-IUD and hysteroscopic treatment offer individualized management strategies, presenting distinct benefits for specific patient subgroups.

By addressing these variables, the present meta-analysis aims to evaluate these two approaches and emphasizes the integration of personalized medicine principles into the treatment of CSDs.

## 2. Materials and Methods

### 2.1. Search Strategy, Eligibility of Studies, and Protocol Registration

The present meta-analysis was performed in accordance with the guidelines for Systematic Reviews and Meta-analyses (PRISMA) based on the authors’ predetermined inclusion criteria [11]. Since all the studies were extracted from previously published data, Institutional Review Board approval was not requested. Selection of abstracts was conducted by two authors (A.D., K.K.) who independently searched the literature. Only studies published in languages using the Latin alphabet were included. The inclusion of studies was based on pre-established eligibility criteria. All observational comparative studies evaluating symptomatic outcomes between patients treated for isthmocele with hysteroscopy and those treated with the insertion of an LNG-IUS were included. Case reports, small case series, letters to the editor, animal studies, and review articles were not included. Conference proceedings and abstracts were also planned to be excluded, as they lack important information that is necessary for the assessment of study limitations and quality of evidence.

The PICO criteria that were used to develop our search strategy were as follows:

- Population: Women undergoing treatment for symptomatic isthmocele.
- Intervention: Insertion of a levonorgestrel-releasing intrauterine system for symptomatic treatment.
- Comparator: Application of hysteroscopy using a resectoscope for symptomatic treatment.
- Outcomes: Postoperative outcomes at follow-up intervals of ≤3 months, 6 months, 9 months, and 12 months (effectiveness rate, total bleeding days per cycle, reduced spotting days per cycle, amenorrhea rate).

The study’s protocol was registered in Open Science Framework prior to the conduct of this review (https://doi.org/10.17605/OSF.IO/H4SN5).

### 2.2. Literature Search and Data Extraction

We used the Medline (1966–2023), Scopus (2004–2023), Google Scholar (2004–2023), Cochrane CENTRAL Register of Controlled Trials, and Clinicaltrials.gov databases in our primary search along with the reference lists of electronically retrieved full-text papers (snowballing). The date of last search was set at 1 August 2024. The search strategy included a combination of the following search terms words: (“Cesarean Section Scar” [MeSH Terms] OR “cesarean scar defect” [All Fields] OR “isthmocele” [All Fields] OR “niche” [All Fields]) AND ((“Hysteroscopy” [MeSH Terms] OR “Hysteroscopic Surgical Procedures” [MeSH Terms] OR “hysteroscopic resection” [All Fields] OR “resectoscopic treatment” [All Fields] OR “hysteroscopic surgery” [All Fields]) OR (“Levonorgestrel” [MeSH Terms] OR “Intrauterine Devices, Medicated” [MeSH Terms] OR “levonorgestrel IUD” [All Fields] OR “LNG-IUS” [All Fields] OR “levonorgestrel-releasing intrauterine system” [All Fields])).

**Figure 1:**
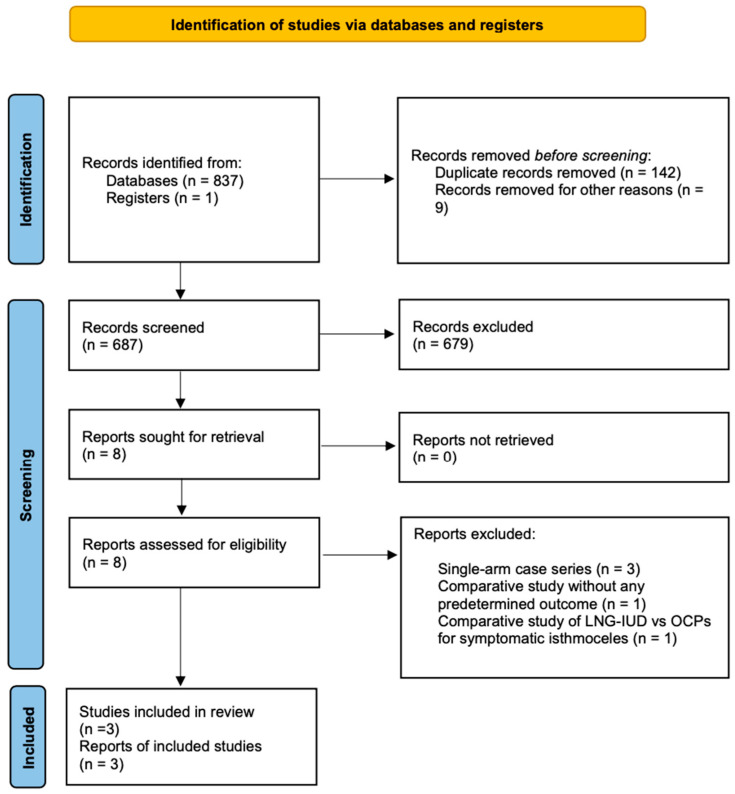
Flow diagram of the detailed process of selection of articles for inclusion in the systematic review and meta-analysis.

The initial selection of studies was conducted based on the titles, followed by an assessment of abstracts when eligibility was uncertain. After eliminating duplicates, the studies were evaluated according to the predefined inclusion and exclusion criteria. Articles that met or appeared to meet these criteria were retrieved for further analysis. Two authors (A.D. and N.K.) independently conducted a comprehensive literature search, resolved redundancies, and organized the selected indices in structured forms. Any discrepancies among the authors were discussed collectively until a consensus was achieved. The PRISMA flow diagram schematically presents the stages of article selection (Figure 1).

### 2.3. Definitions and Predetermined Outcomes

For the purposes of the present study, amenorrhea refers to the absence of menstruation for three consecutive cycles [12,13]. Similarly, the effectiveness rate, in a specific follow-up interval, indicates the proportion of patients who experienced a reduction of 50% or more in the number of spotting days per cycle relative to the total number of patients who received an intervention. As postmenstrual spotting we described the intermenstrual spotting lasting for 2 days, immediate brownish discharge for 2 days after menstruation, or irregular bleeding where the total bleeding days (including both menstrual and spotting days) exceeded 9 days [14]. Lastly, total bleeding days per cycle was defined as the sum of menstruation days and postmenstrual spotting days per cycle.

The primary outcomes assessed in our study were the effectiveness rate, the total bleeding days per cycle, and the reduced spotting days per cycle during the 6- and 12-month follow-up periods. Secondary outcomes were determined following data extraction that was performed using a modified data form based on Cochrane’s data collection form for intervention reviews for RCTs and non-RCTs. These included the aforementioned outcomes during follow-up periods of ≥3 months, as well as at the 9-month follow-up. Additionally, as secondary outcomes, we defined patients’ post-treatment satisfaction, assessed by the five-point Likert scale, and the pooled weighted amenorrhea rate observed in the levonorgestrel IUD group of patients.

### 2.4. Quality Assessment

The Risk of Bias in Nonrandomized Studies of Interventions (ROBINS-I) tool was employed to assess the quality of nonrandomized studies [15]. Randomized controlled trials (RCTs) were evaluated using the Risk of Bias (RoB-2) tool [16]. The ROBINS-I tool examines seven domains of bias in nonrandomized studies: confounders, participant selection, intervention classification, intervention deviations, missing data, outcome measurement, and result reporting. It classifies studies into four levels of bias: low, moderate, serious, and critical. The RoB-2 tool for randomized studies evaluates five domains: the randomization process, intervention deviations, missing outcome data, outcome measurement, and result reporting, categorizing studies into three levels of bias: low risk, some concern, and high risk. Two authors (A.D. and A.M.) independently conducted the quality assessments, with any disagreements resolved by a third author (A.P. (Anastasia Prodromidou)).

### 2.5. Statistical Analysis

This systematic review and meta-analysis were conducted in accordance with the recommendations of the Cochrane Collaboration, as outlined in the Cochrane Handbook for Systematic Reviews of Interventions and following the Preferred Reporting Items for Systematic Reviews and Meta-Analysis (PRISMA) guidelines [17]. Statistical meta-analysis was performed using the RevMan 5.4 software (The Nordic Cochrane Centre, The Cochrane Collaboration, Copenhagen, Denmark, 2020). Confidence intervals were set at 95%. Two authors (A.D. and A.P. (Anastasia Prodromidou)) independently conducted all analyses, with any disagreements resolved by a third author (A.M.). For dichotomous outcomes, odds ratios (ORs) with 95% confidence intervals (CIs) were used to compare pooled results. For continuous outcomes, mean differences (MDs) with 95% CIs were employed. For studies reporting results in formats other than (mean ± standard deviation), conversions were applied, and skewness detection was conducted. Heterogeneity was assessed using Cochran’s Q test and I^2^ statistics, considered significant if *p* < 0.10 or I^2^ > 25%, respectively. Given the anticipated high heterogeneity in the methodological characteristics of included studies, the DerSimonian-Laird random-effects model (REM) was utilized for all comparisons. In estimating weight, the generic inverse-variance method was employed. This method incorporates the standard error and the intervention effect, aggregating data across all studies to provide an estimate. It assumes that variability in effect sizes across studies is due to both sampling errors and inherent differences in effect sizes among studies [18]. For studies reporting median values and ranges, the formula proposed by Hozo et al. was used to estimate the mean and variance (standard deviation) [19]. The cutoff for statistical significance was set at *p* < 0.05.

## 3. Results

### 3.1. Study Selection and Characteristics

Our search strategy, depicted in Figure 1, resulted in 838 abstracts/manuscripts.

Among these, 142 were identified as duplicates across databases, and 687 were excluded based on title and abstract analysis due to irrelevance. A detailed review of eight studies was performed by two authors (A.D. and N.K.), resulting in the exclusion of five studies. Among them, the study by Zhang et al., although a comparative study, included a group of only five patients treated with a levonorgestrel-releasing intrauterine device for isthmocele symptoms. Additionally, it did not include any of the predetermined outcomes of the present study and was therefore excluded [10]. Similarly, the studies by Chen et al., Gencer et al., and Ou et al. were excluded as they consist of small case series of patients whose isthmocele symptoms were treated with the application of LNG-IUD [8,9,20]. Finally, the study by Zheng et al. compared the application of LNG-IUD with the administration of oral contraceptives for the treatment of uterine niche symptoms, and therefore, it was not included in our study [21].

Ultimately, three comparative studies (comprising one randomized controlled trial, one prospective cohort study, and one retrospective study) fulfilled all inclusion criteria and were incorporated into the analysis [22,23,24]. Conducted in China and Taiwan, these studies included a total of 344 patients. Among them, 171 patients (49.7%) received LNG-IUD treatment and the rest were treated with operative hysteroscopy for isthmocele symptoms. Table 1 provides a brief overview of the methodological characteristics of the included studies.

Baseline characteristics that were pre-established as essential for inclusion in the present meta-analysis were under-reported among studies that were finally included. Based on the available data, no statistically significant differences were observed between patients treated for abnormal uterine bleeding symptoms due to isthmocele with operative hysteroscopy and those treated with a levonorgestrel-releasing intrauterine device. In fact, no differences were noted in either the patients’ or the isthmocele characteristics between the two groups. The analyzed indices were tabulated in Table 2.

The RCT included in this systematic review demonstrated a low risk of bias. The two non-RCT studies included in the present meta-analysis exhibited a moderate risk of bias due to confounding. Additionally, the study by Huang et al. also showed a moderate risk of bias in all other parameters, except for bias due to deviations from intended interventions. Assessment of the methodological heterogeneity with the RoB-2 and ROBINS-I tools revealed that the overall quality of analyzed evidence was moderate-high. The detailed assessment of each included study is shown in Table 3.

### 3.2. Outcomes

**Figure 2:**
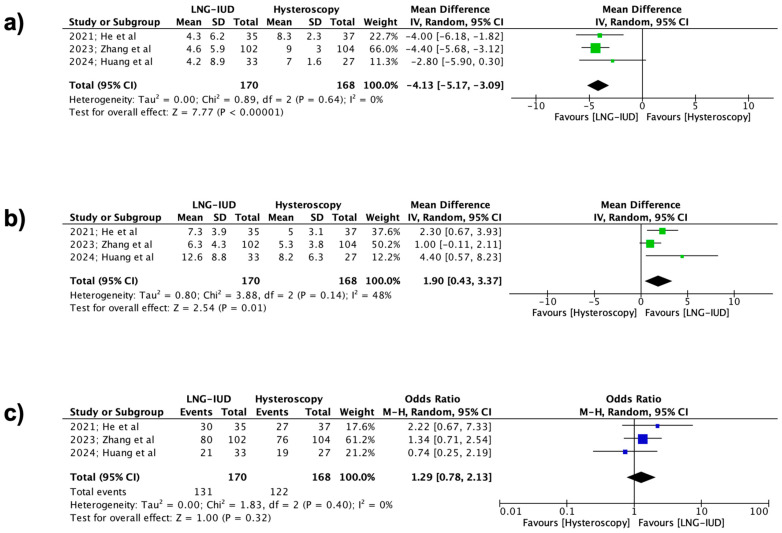
Forest plots describing the contrast between the LNG-IUD group and the hysteroscopic treatment group (6 months follow-up). (**a**) Mean total number of bleeding days per cycle, (**b**) mean reduction in spotting days per cycle, and (**c**) effectiveness rate. (Vertical line = “no difference” point between the two groups. Blue squares = odds ratios; green squares = mean differences; diamonds = pooled mean differences/odds ratios and 95% confidence intervals for all studies; horizontal lines = 95% CI) [22,23,24].

The primary outcomes of the present meta-analysis were the effectiveness rate, the total bleeding days per cycle, and the reduced spotting days per cycle during the 6- and 12-month follow-up periods among the two groups. Specifically, at the 6-month follow-up, a statistically significant difference was observed in favor of the LNG-IUD treatment regarding both the total number of bleeding days per cycle (338 patients, REM, MD −4.13 days, 95% CI: −5.17 to −3.09; *p* < 0.00001) and the mean reduction in spotting days per cycle (338 patients, REM, MD 1.90 days, 95% CI: 0.43 to 3.37; *p* = 0.01). Unfortunately, the same results were not observed concerning the effectiveness rate, where no statistically significant difference was noted between the two groups (338 patients, REM, OR 1.29, 95% CI: 0.78 to 2.13; *p* = 0.32) (Figure 2).

**Figure 3:**
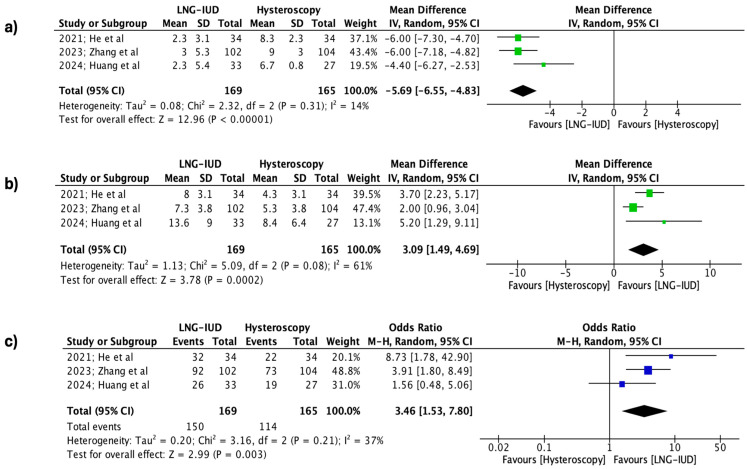
Forest plots describing the contrast between the LNG-IUD group and the hysteroscopic treatment group (12 months follow-up). (**a**) Mean total number of bleeding days per cycle, (**b**) mean reduction in spotting days per cycle, and (**c**) effectiveness rate. (Vertical line = “no difference” point between the two groups. Blue squares = odds ratios; green squares = mean differences; diamonds = pooled mean differences/odds ratios and 95% confidence intervals for all studies; horizontal lines = 95% CI) [22,23,24].

Regarding the differences between the two groups at the 12-month follow-up, it was observed that for all the outcomes, the application of LNG-IUD was superior to the treatment of isthmocele using operative hysteroscopy. Specifically, the application of the levonorgestrel IUD demonstrated statistically significant superiority in the effectiveness rate (334 patients, REM, OR 3.46, 95% CI: 1.53 to 7.80; *p* = 0.003), the reduced spotting days (334 patients, REM, MD 3.09 days, 95% CI: 1.49 to 4.69; *p* = 0.0002), and the total number of bleeding days per cycle (334 patients, REM, MD −5.69 days, 95% CI: −6.55 to −4.83; *p* < 0.00001), respectively (Figure 3).

Finally, all the secondary outcomes, which include the comparison of the two groups for the aforementioned parameters at ≤3 months and 9 months follow-up, patients’ post-treatment satisfaction assessed by the five-point Likert scale at each follow-up interval, and the pooled weighted amenorrhea rate, are briefly summarized in Table 4.

## 4. Discussion

### 4.1. Principal Findings

The results of present meta-analysis reveal that the use of LNG-IUD, compared to hysteroscopic resection for treating isthmocele, leads to a statistically significant reduction in total bleeding days per cycle and a significant increase in reduced spotting days per cycle starting from the 6-month follow-up. Furthermore, patients’ satisfaction in the LNG-IUD group is notably higher from the 6-month follow-up onwards. Crucially, these differences remain consistent through to the 12-month follow-up. Similarly, from 9 months post-intervention, the post-treatment effectiveness, as defined in this study, is superior in the LNG-IUD group, with this advantage maintained at the 12-month follow-up.

### 4.2. Comparison with Existing Literature

Pharmacological management, including oral contraceptives (OCPs) and levonorgestrel-releasing intrauterine devices, have been explored as non-invasive treatment options for cesarean scar defects. OCPs have demonstrated efficacy in managing CSD-associated symptoms such as abnormal uterine bleeding and pelvic pain. The initial studies by Tahara et al. and Florio et al., which addressed the use of contraceptives for managing isthmocele-related bleeding symptoms, reported significant improvements in intermenstrual bleeding and reductions in the duration of postmenstrual spotting through the modulation of endometrial vascularization and hormonal regulation [25,26]. Furthermore, the study by Zheng et al. evaluated the effectiveness of OCPs in managing intermenstrual bleeding caused by uterine niche. Their results demonstrated that the effectiveness of OCPs exceeded 80% at 3 months and 90% at 6 months post-treatment. Specifically, at 6 months, 73% of patients experienced menstrual periods lasting less than 7 days, while only 7% showed no response to treatment. Despite these encouraging results, the benefits of OCPs appear to be primarily symptomatic and transient, as their discontinuation often leads to symptom recurrence. This limits their applicability as a standalone long-term solution [21].

LNG-IUDs have similarly shown to be a promising treatment in reducing bleeding symptoms associated with CSDs. Chen et al. reported an 88.3% efficacy rate in resolving intermenstrual bleeding, attributed to localized effects on endometrial thinning and stromal atrophy [8]. Other studies, such as those by Gencer et al. and Zheng et al., demonstrated reductions in spotting duration and improved bleeding patterns among patients using LNG-IUDs. However, irregular spotting and the persistence of structural defects limit the broader application of LNG-IUDs as a definitive treatment [20,21].

### 4.3. Clinical Implications

**Figure 4:**
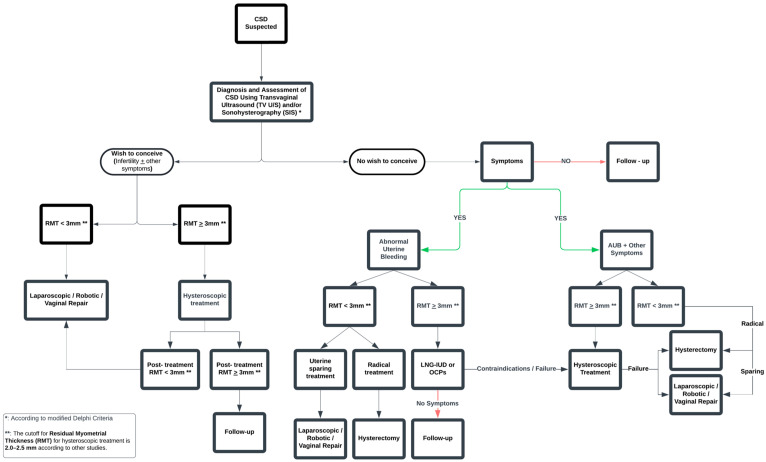
Proposed algorithm for the treatment of patients with cesarean scar defects.

This study represents a systematic endeavor to evaluate pharmacological treatment as a solution for isthmocele symptoms. The objective of our meta-analysis is to catalyze further comprehensive and higher-quality research, which will substantiate or challenge our findings. Based on our findings, we propose a treatment algorithm for managing patients with CSDs, as illustrated in Figure 4. The proposed treatment algorithm, grounded in the principles of personalized medicine, provides practical guidance for selecting the most suitable therapeutic option. According to our algorithm, LNG-IUD is recommended for patients with abnormal uterine bleeding due to cesarean scar defect who do not wish to conceive. Its use is considered a less invasive option compared to other treatment methods. Our recommendation for its use in patients with RMT ≥ 3 mm (or ≥2.5 mm according to others) is based on the fact that, to date, no data are available comparing LNG-IUD to laparoscopic or vaginal repair. Existing studies evaluate LNG-IUD only against hysteroscopic management. Additionally, in our meta-analysis, the LNG-IUD groups across all included studies had an average RMT greater than 2.0 mm. Therefore, any conclusion should be based on the fact that the effectiveness of the LNG-IUD has been studied as an alternative to hysteroscopic treatment, and thus, it should only be recommended as a treatment option only in such cases. Similarly, according to our proposed algorithm, hysteroscopic resection should be considered in cases of failure, contraindications, side effects, or device expulsion. Women should be thoroughly informed prior to the insertion of the LNG-IUD about the expected improvement in abnormal uterine bleeding symptoms, which typically becomes apparent after the first 3 months, as highlighted by the findings of our meta-analysis. Moreover, they should be made aware of the nearly 50% likelihood of developing amenorrhea within 12 months of insertion, enabling them to make a well-informed decision regarding their treatment. Finally, there are no existing data regarding LNG-IUD’s efficacy in managing other CSD-related symptoms such as pelvic pain and dysmenorrhea. However, given its proven positive effects on these symptoms in conditions such as adenomyosis and endometriosis, its use could be explored in future research for patients with similar symptoms caused by CSD [27,28,29].

### 4.4. Strengths and Limitations of the Study

The strengths of this meta-analysis lie in its robust methodological design and originality. By strictly adhering to PRISMA guidelines and registering the protocol in PROSPERO, the study ensures transparency and credibility. Another notable strength is the inclusion of studies without date restrictions, allowing for a comprehensive and thorough data collection process. The extensive search across multiple databases, coupled with independent review by multiple assessors, reinforces the methodological rigor and enhances the reliability of the findings. Furthermore, the clearly defined inclusion criteria, based on the PICO framework, ensure the relevance of the results. Importantly, this study is the first to compare LNG-IUD and hysteroscopic management for symptomatic isthmocele, addressing a significant yet underexplored treatment alternative.

Despite its strengths, this meta-analysis has certain limitations that warrant consideration. The small number of included studies restricts the generalizability of the findings and highlights the need for further research in this area. Moreover, all included studies were conducted in Asia, introducing potential geographic bias and limiting the applicability of the results to more diverse populations. The lack of long-term follow-up data beyond 1 year is another limitation, as it prevents a comprehensive understanding of the sustained effectiveness of the interventions. Inconsistencies in reporting key variables, such as residual myometrial thickness, further constrain the depth of the analysis. Additionally, some degree of heterogeneity was observed in certain outcomes, reflecting the influence of uncontrolled confounding factors across the included studies. While our analysis was limited by the available data, future research incorporating larger datasets could benefit from advanced statistical methods, such as meta-regression or subgroup analysis, to better explore the sources of variability and identify patient populations that may derive the most benefit from each treatment approach. These factors underscore the importance of future high-quality studies to validate and expand upon these findings.

## 5. Conclusions

In conclusion, the present meta-analysis sheds light on the potential benefits of LNG-IUD compared to hysteroscopic resection for symptomatic isthmocele, offering promising initial insights. The adoption of personalized therapeutic approaches, such as the LNG-IUD for women with CSD with abnormal uterine bleeding, who do not desire future pregnancies and meet the RMT criteria, represents a fundamental step toward improving patient care. However, the evidence remains preliminary, with limited studies and short-term follow-up data. While the findings suggest LNG-IUD as a viable non-invasive alternative, further high-quality research with larger, more diverse populations, and extended follow-up is essential to confirm these results and establish robust clinical guidelines.

## Figures and Tables

**Table 1:** Methodological characteristics of the included studies (LNG-IUD vs. operative hysteroscopy).

Year; Author	Study Design	Country	Study Period	Patient No.	Inclusion Criteria	Exclusion Criteria
2021; He et al. [22]	PCS	China	April 2018– April 2019	36 vs. 38	Patients aged between 20 and 45 years, diagnosed with a niche with postmenstrual spotting of at least 2 days, who considered to undergo a hysteroscopic niche resection or to receive a LNG-IUD (52 mg).	Fertility wish within 1 year, irregular menstrual cycle before the last cesarean delivery, IUD present, length of uterine cavity < 6 cm or >10 cm, coagulopathy, current exogenous hormone treatment, and other gynecological conditions that could cause prolonged bleeding such as leiomyoma, endometrial hyperplasia, ovarian endometriosis, and pregnancy. Moreover, patients had received hysteroscopic resection or LNG-IUD before intervention.
2023; Zhang et al. [24]	RCT	China	September 2019– January 2022	102 vs. 104	Women with postmenstrual spotting after CD, with a niche in the anterior wall of the LUS of at least 2 mm in depth and a residual myometrium of at least 2.2 mm on MRI, aged 18 to 48 years, and without a desire to conceive within 1 year.	Contraindications or unwillingness for either hysteroscopic niche resection or LNG-IUD placement; positive pregnancy test; current use of an intrauterine device; contraindications to general or local anesthesia; a history of coagulation disorder or high risk for anticoagulant use; malignancy, endometrial polyp, atypical endometrial cells, cervical dysplasia, or hydrosalpinx that may communicate with the uterus; presence of adenomyosis, submucosal leiomyoma, or leiomyoma causing uterine cavity length 9 cm assessed by transvaginal ultrasound or MRI; endocrine disorders resulting in changes in the menstrual cycle; and menstrual cycle disorders (>35 days or intercycle variation of 2 weeks).
2024; Huang et al. [23]	RS	Taiwan	April 2013– November 2021	33 vs. 26	Patients diagnosed with niche by hysteroscopy who received either resectoscopic remodeling procedures or LNG-IUD insertion for intermenstrual bleeding treatment.	Patients with pelvic inflammatory disease, vaginitis, cervical infection, submucosal myoma, or other endometrial lesions.

Abbreviations: **LNG-IUD**: levonorgestrel intrauterine device; **LUS**: lower uterine segment; **CD**: cesarean delivery; **PCS**: prospective cohort study; **RCT**: randomized controlled trial; **RS**: retrospective study.

**Table 2:** Patients’ and isthmocele characteristics (LNG-IUD vs. operative hysteroscopy).

	2021; He et al. [22]	2023; Zhang et al. [24]	2024; Huang et al. [23]	*p*-Value
**Patients’ Characteristics**				
**Age (years)**	34.9 ± 4.1 vs. 34.4 ± 4.1 **^a^**	36.4 ± 3.9 vs. 36.8 ± 4.5 **^a^**	41.7 ± 5.2 vs. 39.5 ± 4.4 **^a^**	0.52
**Gravidity**	3 ± 1.54 vs. 2 ± 1.54 **^a^**	2.3 ± 0.75 vs. 2.3 ± 0.75 **^a^**	NRD	0.37
**Parity**	1.7 ± 0.8 vs. 1.3 ± 0.8 **^a^**	1.7 ± 0.8 vs. 1.7 ± 0.8 **^a^**	2.1 ± 0.6 vs. 2.5 ± 0.7 **^a^**	0.98
**Number of CS**	1.3 ± 0.8 vs. 1.3 ± 0.8 **^a^**	1.7 ± 0.8 vs. 1.7 ± 0.8 **^b^**	2.0 ± 0.6 vs. 2.1 ± 0.8 **^a^**	0.81
**Time since last CS (years)**	NRD	5.9 ± 4.2 vs. 6.2 ± 4.2 **^a^**	6.85 ± 8.95 vs. 2.63 ± 4.6 **^a^**	0.46
**Uterine and Isthmocele Characteristics**				
**Retroflexion rate (%)**	19/36 (53%) vs. 16/38 (42%)	25/102 (24.5%) vs. 22/104 (21.2%)	11/33 (33.3%) vs. 4/27 (15.4%)	0.12
**RMT (mm)**	3 ± 1.5 vs. 4.7 ± 2.3 **^a^**	3.8 ± 1.5 vs. 3.8 ± 1.5 **^a^**	4.3 ± 1.95 vs. 6.1 ± 1.96 **^a^**	0.10
**Length (mm)**	6 ± 1.54 vs. 5 ± 1.54 **^a^**	7.5 ± 2.7 vs. 7.5 ± 3.5 **^a^**	NRD	0.29
**Width (mm)**	11.7 ± 3.1 vs. 11.3 ± 3.85 **^a^**	15.4 ± 4.8 vs. 14.4 ± 4.3 **^a^**	NRD	0.12
**Depth (mm)**	6 ± 1.5 vs. 4.7 ± 2.3 **^a^**	6.2 ± 1.6 vs. 5.1 ± 1.6 **^a^**	NRD	**<0.001**

Abbreviations: **a**: mean ± standard deviation; **RMT**: residual myometrial thickness; **NRD**: no reported data; **CS**: cesarean section; bold values indicate statistically significant differences

**Table 3:** Risk of bias of the included studies (risk of bias summary).

Study	Type	Tool	Bias Arising from the Randomization Process	Bias in Selection of Participants into the Study	Bias in Classification of Interventions	Bias Due to Confounding	Bias Due to Deviations from Intended Interventions	Bias Due to Missing Data	Bias in Measurement of Outcomes	Bias in Selection of the Reported Result	Overall Risk of Bias
**2021; He et al.** [22]	Non-RCT	ROBINS-I	**X**	Low Risk	Low Risk	Moderate Risk	Low Risk	Low Risk	Low Risk	Low Risk	Moderate
**2023; Zhang et al.** [24]	RCT	RoB-2	Low Risk	**X**	**X**	**X**	Low Risk	Low Risk	Low Risk	Low Risk	Low
**2024; Huang et al.** [23]	Non-RCT	ROBINS-I	**X**	Moderate Risk	Moderate Risk	Moderate Risk	Low Risk	Moderate Risk	Moderate Risk	Moderate Risk	Moderate

Abbreviations: **RCT**: randomized controlled trial; **RoB-2**: Risk of Bias 2; **ROBINS-I**: Risk of Bias In Non-randomized Studies—of Interventions.

**Table 4:** Secondary outcomes of the meta-analysis (LNG-IUD vs. operative hysteroscopy).

Outcome	Number of Studies	Sample Size	Meta-Analytic Model	Pooled Outcome (MD, OR, Pooled Weighted Rate)	95% CI	*p*-Value	I^2^
**≤3 Months Follow-up**							
Effectiveness Rate	3 studies	170 vs. 169	REM	(OR) 1.31	0.64 to 2.69	0.45	48%
Reduced Spotting Days per Cycle	3 studies	170 vs. 169	REM	(MD) −0.40	−1.50 to 0.70	0.48	0%
Total Bleeding Days per Cycle	3 studies	170 vs. 169	REM	(MD) −0.78	−2.33 to 0.78	0.33	0%
Post-treatment Satisfaction (five-point Likert Scale)	2 studies	137 vs. 142	REM	(MD) 0.25	−0.61 to 1.11	0.57	71%
**6 Months Follow-up**							
Post-treatment Satisfaction (5-point Likert scale)	2 studies	137 vs. 141	FEM	(MD) 0.37	0.09 to 0.66	**0.009**	0%
**9 Months Follow-up**							
Effectiveness Rate	2 studies	123 vs. 99	FEM	(OR) 3.72	1.88 to 7.35	**0.0002**	0%
Reduced Spotting Days per Cycle	2 studies	136 vs. 138	FEM	(MD) 2.23	1.34 to 3.12	**<0.00001**	0%
Total Bleeding Days per Cycle	2 studies	136 vs. 138	FEM	(MD) −4.83	−5.80 to −3.86	**<0.00001**	0%
Post-treatment Satisfaction (5-point Likert Scale)	2 studies	136 vs. 138	REM	(MD) 0.59	0.24 to 0.94	**0.0009**	28%
**12 Months Follow-up**							
Post-treatment Satisfaction (5-point Likert Scale)	2 studies	136 vs. 138	REM	(MD) 0.59	0.24 to 0.94	**0.0009**	28%
Amenorrhea Rate	3 studies	171	FEM	47.4%	46.5 to 48.3	**-**	0%

Abbreviations: **REM**: random effects model; **FEM**: fixed effects model; **MD**: mean difference; **OR**: odds ratio; bold values indicate statistically significant differences.

## Data Availability

Dataset available on request from the authors.
